# Pan-Cancer Analysis Reveals Functional Similarity of Three lncRNAs across Multiple Tumors

**DOI:** 10.3390/ijms24054796

**Published:** 2023-03-01

**Authors:** Abir Khazaal, Seid Miad Zandavi, Andrei Smolnikov, Shadma Fatima, Fatemeh Vafaee

**Affiliations:** 1 School of Biotechnology and Biomolecular Sciences, Faculty of Science, University of New South Wales, Sydney, NSW 2052, Australia; 2 UNSW Data Science Hub, University of New South Wales, Sydney, NSW 2052, Australia; 3 Harvard Medical School, Harvard University, Boston, MA 02115, USA; 4 Ingham Institute of Applied Medical Research, Sydney, NSW 2170, Australia

**Keywords:** long non-coding RNA, cancer, mRNA, pan-cancer analysis, functional analysis, gene ontology

## Abstract

Long non-coding RNAs (lncRNAs) are emerging as key regulators in many biological processes. The dysregulation of lncRNA expression has been associated with many diseases, including cancer. Mounting evidence suggests lncRNAs to be involved in cancer initiation, progression, and metastasis. Thus, understanding the functional implications of lncRNAs in tumorigenesis can aid in developing novel biomarkers and therapeutic targets. Rich cancer datasets, documenting genomic and transcriptomic alterations together with advancement in bioinformatics tools, have presented an opportunity to perform pan-cancer analyses across different cancer types. This study is aimed at conducting a pan-cancer analysis of lncRNAs by performing differential expression and functional analyses between tumor and non-neoplastic adjacent samples across eight cancer types. Among dysregulated lncRNAs, seven were shared across all cancer types. We focused on three lncRNAs, found to be consistently dysregulated among tumors. It has been observed that these three lncRNAs of interest are interacting with a wide range of genes across different tissues, yet enriching substantially similar biological processes, found to be implicated in cancer progression and proliferation.

## 1. Introduction

Cancer is a complex disease that continues to be a health burden globally [1]. It is characterised by dynamic genomic alterations, including somatic mutations, epigenetic modifications, copy number variations, and changes in expression profiles [2,3,4]. The emergence of massively parallel sequencing technologies has enabled systematic documentation of the genetic changes in tumors and introduced the concept of the cancer genome [5,6,7]. Considered a landmark in cancer genomics, The Cancer Genome Atlas (TCGA) program has produced, to date, more than 2.5 PB of multi-layered omic data, along with clinical profiles for more than 11,000 patients across 33 cancer types [8,9,10]. TCGA has improved our understanding of cancer genomics, revolutionised cancer classification and identified therapeutic targets [8,11,12].

Although cancers have their own genetic identity with distinct, tissue-specific changes, many tumors share similar genetic alterations that disrupt common biological processes [13,14]. Emerging computational technologies and rich datasets present an opportunity to explore the differences and similarities of genetic and molecular changes across different tumor types using a set of techniques collectively referred to as pan-cancer analyses [14,15]. The importance of pan-cancer profiling lies in its ability to provide a comprehensive analysis of the genetic changes associated with multiple cancers. In addition, not only does it identify shared patterns that aid in the development of uniform treatments strategies, but also distinguishes those unique alterations and enhances personalised care [14].

With the decreasing cost of whole genome sequencing, there is a growing focus on performing pan-cancer analysis on non-coding regions of the genome. An increasing body of evidence suggests non-coding RNAs (ncRNAs) play an important role in biogenesis of cancer [16]. While some ncRNAs have been well studied, such as microRNAs [17], other types have been studied less extensively, including long ncRNAs (lncRNAs). LncRNAs are transcripts with a length greater than 200 nucleotides, exhibiting similar molecular characteristics to messenger RNAs (mRNAs) but lacking an appreciable potential to code for proteins [18,19]. Localised either in the nucleus or cytoplasm, lncRNAs form a complex network of interactions with DNA, RNA, and proteins [20]. Although it is still debatable whether the majority of lncRNAs are simply transcriptional noise, some have been found to be associated with important biological roles. For example, lncRNA *XIST* is known to initiate silencing of the inactive X chromosome during X inactivation [21,22]. More generally, studies have suggested lncRNAs as cis- and trans-acting regulators of gene expression via chromatin reprogramming [23,24]. They have also been implicated in post-transcriptional regulation, including mRNA translation [25], as well as cell differentiation and development [26]. Despite these findings, lncRNA functions remain poorly understood.

LncRNAs are engaged in many cellular functions, and their dysregulation has been linked to diseases, including cancer [16,27]. Aberrant expression of lncRNAs has been identified in different tumors including brain, breast, and colon cancer [28,29]. Many lncRNAs have also been shown to be regulated by oncogenes and tumor suppressors, suggesting a role in oncogenesis [30]. Furthermore, functional studies have revealed validated cancer roles for more than hundred lncRNAs in tumors [31]. A wide range of cancer treatments are currently available [32,33]; chemotherapy, however, continues to be preferred despite its diminishing effectiveness when cancer has advanced or metastasised [33,34]. Poor prognosis is probably due to late diagnoses of cancer, together with tumors having acquired drug resistance, and continues to be a major challenge in treating malignancies [34]. It is thus important to search for new biomarkers for early diagnosis and therapeutic targets for more effective treatments. A plethora of evidence has revealed dysregulation of lncRNAs to be associated with cell proliferation, apoptosis, invasion and drug resistance, processes found implicated in the pathogenesis of cancer [35,36]. These findings put forward lncRNAs as potential biomarkers and therapy agents.

At present, there is an increasing focus on identifying lncRNAs associated with tumorigenesis and elucidating their functional implications. Rich RNA-seq datasets are a promising tool for this purpose, but their use can be computationally challenging. To highlight the importance of lncRNA association with cancer and overcome these challenges, The Atlas of Noncoding RNAs in Cancer (TANRIC) was developed [37]. TANRIC is a free and interactive database which gives users access to genomic, proteomic, clinical and lncRNAs expression data of 8143 samples (tumorous and non-neoplastic) from TCGA and others.

In order to characterise common, aberrantly expressed lncRNAs we performed a pan-cancer analysis on lncRNA expression profiles from a TCGA derived dataset using TANRIC platform. We hypothesise that those found to be implicated in different cancer types may exhibit similar functional implications across cancers. To assess this hypothesis, we sought to identify dysregulated lncRNAs across eight TCGA cancer types and explored commonality among different malignancies. We then investigated their functional implications, by performing functional analysis and exploiting the similarity of enriched biological processes. Previous pan-cancer studies have focused on somatic mutations of whole genomes [15], tumor microenvironments [38] as well as proteomic profiles [39]. Putative functions have been studied for onco-lncRNAs dysregulated in multiple cancers [40] without focusing on common consistently dysregulated lncRNAs or exploring the similarity of gene ontologies across different cancer types as performed in our study.

## 2. Results

### 2.1. Common Dysregulated lncRNAs

**Figure 1:**
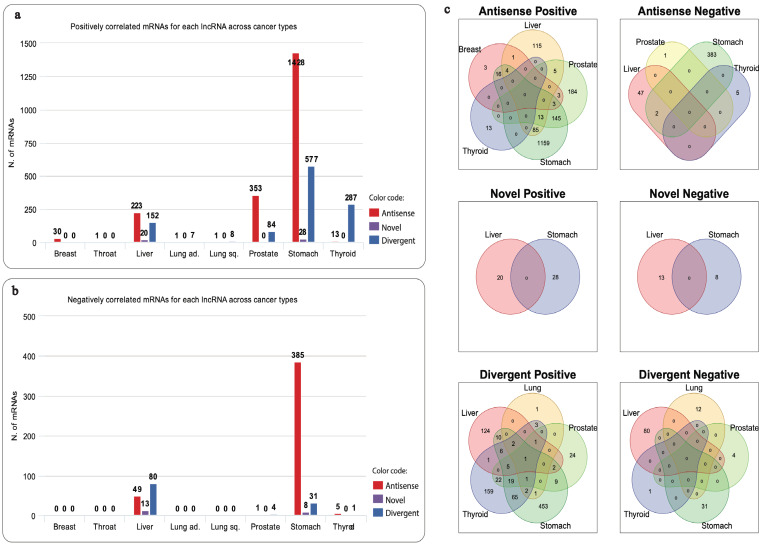
Bar plots showing the number of mRNAs related to the three lncRNAs, per cancer type. Red represents Antisense, purple represents Novel and blue represents Divergent. (**a**) number of positively correlated mRNAs. (**b**) number of negatively correlated mRNAs. (**c**) Venn diagram showing the overlap between lists of positively and negatively correlated mRNAs with lncRNAs: Antisense, Novel and Divergent, across different cancer types: breast (BRCA), thyroid (THCA), stomach (STAD), prostate (PRAD), lung (LUAD and LUSC) and liver (LIHC).

In total, 9616 lncRNAs manifested significant differential expression across cancers (Table S1). Whilst similarity between pairs of cancers, represented by the Jaccard index, appears low (Figure S1), collectively the number of shared lncRNAs of one cancer type with the remaining types is quite high, with overlap ranging from ~80% to ~97% (Table S2). Of the large number of lncRNAs found to be overlapping among different cancer pairs, seven were observed to be differentially expressed in all cancer types (Table S3). Often the same lncRNA deemed upregulated in one cancer type can be found downregulated in another, or the other way around. Nonetheless, three lncRNAs were found to be consistently dysregulated across all cancers: ENSG00000235904 (*RBMS3-AS3*) (hereafter, “Antisense”) and ENSG00000261472 (Novel transcript) (hereafter, “Novel”) are both upregulated, and ENSG00000272455 (*MRPL20-DT*) (hereafter, “Divergent”) is downregulated (Table S4). We chose to focus in the present study on the three consistently dysregulated lncRNAs: Antisense, Novel and Divergent. Correlation analysis revealed in total, 3141 coding genes (|rs| ≥ 0.5 and *p*-value ≤ 0.01), with 2185 mRNAs found to be co-expressed with Antisense, 69 mRNAs with Novel and 1026 mRNAs with Divergent (Figure 1a,b). We found little overlap between correlated mRNAs across different cancers. It appears that for a given lncRNA, the group of co-expressed mRNAs is specific for each cancer type (Figure 1c). The full list of correlated mRNAs can be found in Table S5.

### 2.2. Gene Ontologies (GO) for Inference of Functional Similarity

After identifying and combining GO terms enriched by each lncRNA, additional filtering (FDR ≤ 0.05) resulted in the acquisition of three GO terms lists: Two lists of GO terms associated with Antisense and one list with Divergent, with no records linked with lncRNA Novel (Figure S2). In order to provide functional elucidation of the remaining lncRNAs of interest, we explored similarity between GO term pairs using NaviGO and GO pairwise similarity networks were created (RSS ≥ 0.05).

#### Similarity Networks

**Figure 2:**
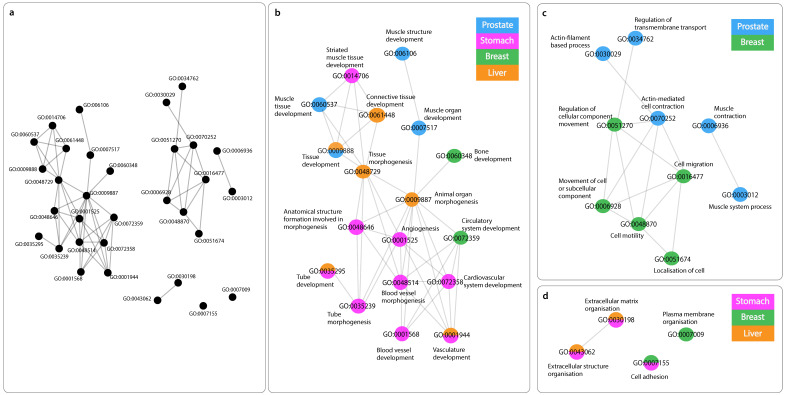
Functional similarity of GO terms associated with mRNAs of positive correlation with lncRNA Antisense. Network visualisation of functional similarity of GO terms enriched by positively correlated mRNAs with lncRNA “Antisense”, across cancers (when applicable, RSS ≥ 0.05). (**a**) Three clusters can be visually identified. (**b**) Close up of cluster 1. (**c**) Close up of cluster 2. (**d**) Close up of cluster 3. (**b**–**d**): Each node indicates a GO term and the edges (lines in between) represent a functional similarity, representing relationships, such as “is a” or “part of”. The color(s) of nodes represent the cancer type(s) in which GO term was found to be enriched, green for breast cancer (BRCA), orange for liver cancer (LIHC), blue for prostate cancer (PRAD) and purple for stomach cancer (STAD.

**Figure 3:**
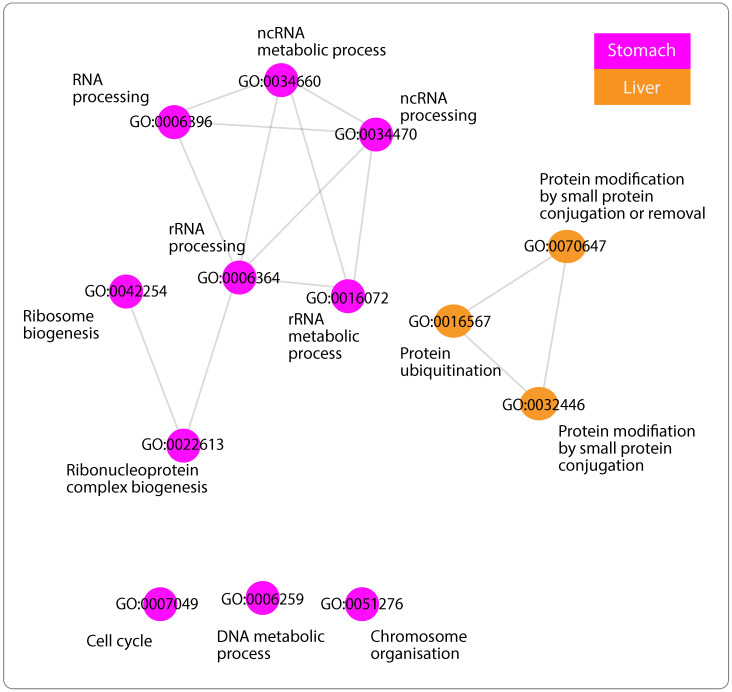
Functional similarity of GO terms associated with mRNAs of negative correlation with lncRNA Antisense. Network visualisation of functional similarity of GO terms enriched by negatively correlated mRNAs with lncRNA “Antisense”, across cancers (when applicable, RSS ≥ 0.05). The color(s) of nodes represent the cancer type(s) in which GO term was found to be enriched, orange for liver cancer (LIHC) and purple for stomach cancer (STAD).

**Figure 4:**
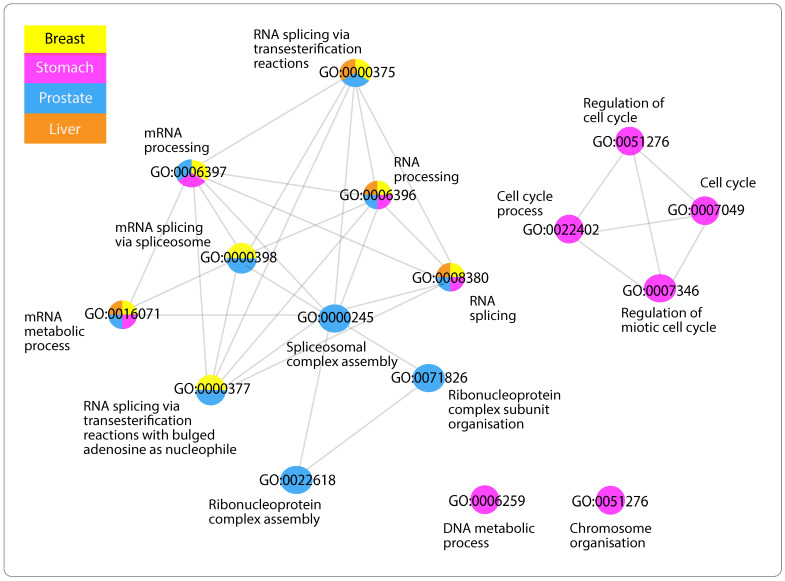
Functional similarity of GO terms associated with mRNAs of positive correlation with lncRNA Divergent. Network visualisation of functional similarity of GO terms enriched by negatively correlated mRNAs with lncRNA “Antisense”, across cancers (when applicable, RSS ≥ 0.05). The color(s) of nodes represent the cancer type(s) in which GO term was found to be enriched, orange for liver cancer (LIHC), blue for prostate cancer (PRAD), purple for stomach cancer (STAD) and yellow for thyroid cancer (THCA).

Starting with GO list enriched by mRNAs positively correlated with Antisense, three clusters in the network of functionally similar GO terms were identified (Figure 2a). The first cluster contains GO terms predominantly involved in tissues and vessel morphogenesis, together with tissues and vasculature development (Figure 2b). The second cluster includes system and cellular processes such as actin-mediated cell contraction in addition to localisation and movement of cell and/or subcellular component (Figure 2c). Finally, GO terms in the third cluster found of extracellular matrix, structure organisation along with cell adhesion (Figure 2d). Network of mRNAs negatively correlated with Antisense, displayed GO terms appear to be mainly associated with ncRNA metabolic processes, particularly ribosomal RNA (rRNA), ribosome biogenesis, and ubiquitination (Figure 3). Lastly, mRNAs positively correlated with Divergent have enriched substantially similar GO terms, immersed with mRNA processing, splicing and metabolism, in addition to processes associated with cell cycle (Figure 4).

**Figure 5:**
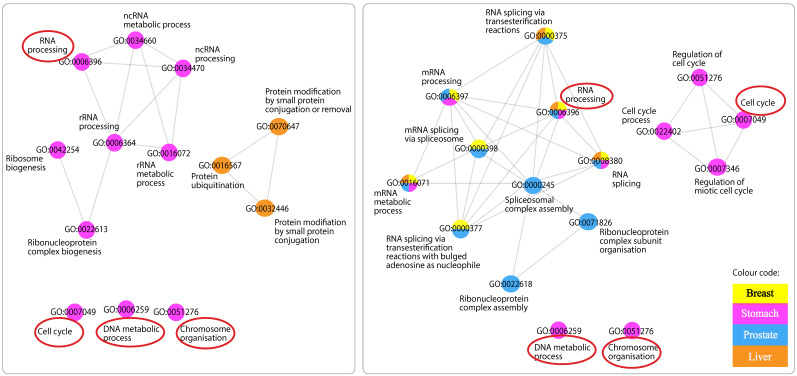
Comparison of Antisense and Divergent GO terms similarity networks. Similarity networks among GO terms enriched by mRNAs related negatively to Antisense (left) and mRNAs related positively to Divergent (right). Shared GO terms in both networks are encircled in red.

Finally, when GO terms enriched by both lncRNAs were compared, high similarity was found between GO terms enriched by mRNAs negatively correlated with Antisense and those enriched by mRNAs positively correlated with Divergent (Figure S3). Interestingly, after further scrutinisation of the different networks, four GO terms were found to be shared between the two: DNA metabolic process, chromosome organisation, cell cycle and RNA processing (Figure 5).

## 3. Discussion

### 3.1. Dysregulation of lncRNAs among Cancers

The identification of 9616 dysregulated lncRNAs suggests pervasive variation of lncRNA expression in cancers, consistent with previous studies [17,41]. Therefore, understanding functional implications of lncRNAs in malignancies is of high importance, as not only can this serve in developing diagnostic tools, but it can also lead to new treatment strategies. Upon exploring commonality of dysregulated lncRNAs, it was observed that tumors share a substantial number of genes (average overlap ~90%), suggesting that potentially the same lncRNAs could be associated with different tumors across different tissues. Whilst it has been suggested that lncRNAs are cancer specific, with tumors of different types and subtypes exhibiting different expression patterns [41,42], some evidence show otherwise. For instance, *MALAT1* was suggested to be involved in multiple tumors; inhibition of the well-studied lncRNA was found to prevent lung cancer metastasis [43]. Conversely, a more recent study showed that knocking out *MALAT1* actually promotes metastasis in breast cancer, suggesting its role as a metastasis suppressant [44]. Additionally, an oncogenic role has also been proposed for *MALAT1* in colorectal carcinoma [45]. Nonetheless, we observed 2855 lncRNAs to be dysregulated uniquely in one cancer type, denoting some level of specificity.

### 3.2. Three Consistently Dysregulated lncRNAs

Intriguingly, three lncRNAs showed striking consistent dysregulation: ENSG00000235904, known as *RBMS3-AS3* gene and ENSG00000261472, a novel transcript, both exhibited upregulation in all tumor samples whilst ENSG00000272455, known as *MRPL20-DT* gene, manifested downregulation.

#### 3.2.1. ENSG00000235904, RBMS3-AS3 “Antisense”

As with the majority of lncRNAs, little is known about the functional implication of *RBMS3-AS3* or its association with tumors. According to lncATLAS [46], *RBMS3-AS3* is found to be expressed mainly in the cytoplasm. Generally, cytoplasmic lncRNAs are believed, through formation of complexes with RNA binding proteins, to be involved in different mechanisms, such as mRNA translation and stability [47,48,49] and protein localisation [50,51]. With regard to cancer, *RBMS3-AS3* has been proposed as a competing endogenous RNA (ceRNA), targeted by several miRNAs in breast cancer [52]. In addition, *RBMS3-AS3* was shown to be serving as miRNA sponge, acting as a tumor suppressor in prostate cancer [53]. We explored TANRIC’s survival analysis for both of these cancer types and found the survival rate across patients to be higher in those who have lower expression of *RBMS3-AS3* (Kaplan–Meier analysis and log-rank test, *p*-value < 0.05). Furthermore, both gastric and colorectal cancers exhibited dysregulation of *RBMS3-AS3* and involvement in ceRNA network [54,55]. Taken together, *RBMS3-AS3* seems to display aberrant expression patterns across tumors; further studies are required to investigate its function and potential involvement in cancer.

#### 3.2.2. ENSG00000261472 “Novel”

Although this lncRNA was also found to be consistently upregulated across tumors, little is known about its association with cancer. ENSG00002614172 is small in size (<500 bp), and unlike *RBMS3-AS3*, no localisation information was found in lncATLAS [46]. Expression across tissues was almost negligible, with body fat having the highest value of only 1.7 TPM, according to the Genotype Tissue Expression project GTEX [56]. Lack of information on this transcript is possibly due to it being newly annotated, and most importantly minimally expressed across tissues. In the literature, we came across a breast cancer analysis where ENSG00000261472 was listed among other enriched lncRNAs [57]. However, to the best of our knowledge, no other studies have been published with reference to cancer. Concisely, ENSG00000261472 is a novel lncRNA whose cellular function is yet to be discovered.

#### 3.2.3. ENSG00000272455, MRPL20-DT “Divergent”

Similar to Novel, no localisation information was detected in lncATLAS for *MRPL20-DT* [46]. A recent study, however, found that *MRPL20-DT* promoter is consistently upregulated across 13 tumors [58]. Likewise, its upregulation amid other lncRNAs was reported in muscle invasive bladder cancer [59]. In contradiction, TANRIC’s survival analysis displayed better survival probability for those with higher expression of *MRPL20-DT* in bladder cancer (Kaplan–Meier analysis and log-rank test, *p*-value < 0.05), which comes in conformity with our results of it being downregulated in malignancy. In essence, *MRPL20-DT* role is still undetermined, but evidence suggest its possible association with cancer. Future research is needed to investigate its dysregulation in tumors and better understand the molecular mechanisms involved.

### 3.3. Specificity of Correlated mRNAs 

Co-expressed mRNAs lists, classified between positively and negatively correlated, ranged in size across different cancers and different lncRNAs, proposing a wide and diverse network of gene interactions across tumors. In addition, the number of correlated mRNAs of a given lncRNA was dependent on the tissue type. For instance, 1428 mRNAs were positively correlated with Antisense in stomach cancer sample set, compared to only 353 and 30 mRNAs in prostate and breast cancer, respectively (Figure 1a), suggesting some level of tissue specificity, which comes in accordance with previous findings [60]. Although there is little intersect between malignancies, mRNAs were noted to be predominantly different (Figure 1c), suggesting that, despite the commonality of the three lncRNAs of interest, they appear to be interacting with different mRNAs in different tissues. Taken together, lncRNAs seem to manifest both tissue-specific and ubiquitous relations, interacting with a broad range of genes across tumors.

### 3.4. Enriched GO Terms

GO enrichment analysis differentiated three GO term lists; no GO terms were found to be notably associated with Novel post-filtration, possibly because the number of correlated mRNAs was the lowest compared to Antisense and Divergent, hence, as a consequence no significant ontology enrichment was detected. The absence of GO terms is somewhat surprising but does not undermine the possible involvement of this lncRNA with tumors. It is worth noting that there is evidence of enrichment of Novel in breast cancer [57], in addition to the present study where consistent upregulation was outlined across all eight cancer types. These initial findings are promising; further studies would make a worthwhile contribution, to better understand the underlying mechanisms related to cancer. 

### 3.5. Networks of Functionally Similar GO Terms

In order to understand the functional similarities of the two remaining lncRNAs, Antisense and Divergent, we identified three GO similarity networks. It is worth noting that the scoring scheme (RSS) we adopted in creating these networks showed minimal variation when compared with Resnik and Lin’s semantic similarity, another two widely used and known measures [61,62]. Whilst this increases our confidence with results presented here, a caveat with this approach is that network representation differs slightly based on the cut-off used with these scoring schemes. With that said, this is generally the case with many analyses and statistical tests which rely on arbitrary cut-off values, and in our analysis, cut-off value does not change the number or nature of biological processes involved, rather the way they are represented in a network.

#### 3.5.1. GO Terms of Positively Related mRNAs with “Antisense”

Three clusters can be identified in this network (Figure 2a). The first cluster comprised of biological processes linked to angiogenesis, blood vessel and tissue morphogenesis as well as vasculature development, all known to be critical for cancer growth (Figure 2b). For instance, it is well-established that angiogenesis is one of the hallmarks of cancer [3]; tumor cells recruit new blood vessels to allow for nutrients and oxygen delivery, as well to be able to metastasise to other tissues [63,64], and this also involves the development of new blood vessels and tissues. This is of importance as it implies that Antisense might be involved with pivotal mechanisms of tumorigenesis. Simultaneously, the second cluster encompassed GO terms of cell motility and migration along with actin filament-based processes (Figure 2c). These processes are also linked to those seen in the first cluster; taking cell motility for example, this is essential in allowing tumor cells to enter the vasculature, transport through blood vessels and invade other sites [65]. Moreover, networks of actin protein filaments form actin cytoskeleton, involved primarily in cell migration and motility in cancer, leading to metastasis [66,67]. Finally, the third cluster involved extracellular matrix and structure organisation together with cell adhesion, processes also associated with cancer progression (Figure 2d). Indeed, there has been a focus on understanding the dysregulation of the extracellular matrix in complex diseases such as cancer. Being the major component of the tumor ‘microenvironment’, it has been suggested to modulate cell behavior and influence adhesion and migration of cells [68,69]. Collectively, the GO terms presented in this network appear to be closely related, describing vital processes for the proliferation and progression of malignancies. The question remains though, whether Antisense is exerting a regulatory role in this network or is simply a by-pass product. Further investigation is required to understand its potential role.

#### 3.5.2. GO Terms of Negatively Related mRNAs with “Antisense”

The smaller list of negatively correlated mRNAs with Antisense enriched important functions relating to ncRNA metabolic processes, particularly rRNA, ribosome biogenesis, and ubiquitination (Figure 3). Mounting evidence suggests that impaired ribosomal activity drives tumorigenesis [70,71]. Ribosome biogenesis is an important regulator of cellular activities, including cell growth and cell cycle progression [72,73]; an increase in rRNA processing is observed during G1 of interphase, in preparation for protein translation, whilst during mitosis, downregulation of ribosomal activity is needed to signal the ending of cell cycle. Uncontrolled cell proliferation, a common feature in cancer, is a consequence of impaired ribosomal activity. Furthermore, it is now believed that perturbation of ribosomal biogenesis is sufficient to lead to malignant transformation [74]. Another process found is ubiquitination (also known as ubiquitylation), a post-translational mechanism in which proteins are tagged by the conjugation of ubiquitin, for modification. Ubiquitin is a small regulatory protein that is highly conserved in eukaryotes, most commonly found to initiate proteins degradation, apart from also altering protein–protein interactions and modulating cellular processes such as cell cycle, apoptosis cell signaling and DNA repair [75,76]. It has been shown that cytoplasmic lncRNAs interfere with protein expression by either obstructing or promoting ubiquitination [77]. With a balanced ribosomal genesis for instance, tumor suppressor protein p53 is usually post-translationally downregulated through ubiquitination. However, studies have revealed that disruption of ribosomal biogenesis primarily causes activation of p53, and consequently disrupts its degradation through ubiquitination [70]. Hence, it comes as no surprise that these processes are interconnected, and their perturbation is associated with carcinogenesis. lncRNA Antisense, identified in this study, has been shown to be associated with a range of important biochemical processes, explicitly ubiquitination. Currently, novel strategies are being developed to target certain pathways in which ubiquitin is primarily involved, resulting in potentiation of drug efficacy, and overcoming multi drug resistance [78].

#### 3.5.3. GO Terms of Positively Related mRNAs with “Divergent”

Finally, the network similar GO terms related with “Divergent” displayed processes, enriched in more than one cancer type, relating to mRNA processing, in particular, splicing pathways in addition to cell cycle regulation, specifically mitosis (Figure 4). Accounting primarily for protein diversity, splicing is a fundamental step of mRNA processing where the same coding gene can have different, even at times, opposing functional transcripts called isoforms. It has been revealed that aberrant splicing is linked with cancer, yielding cancer-specific isoforms favorable for tumor growth [79,80]. In addition, defective splicing also perturbs the cell cycle, which is also enriched in this network. Consistent with the literature, lncRNAs have been suggested to influence splicing in diseases such as cancer [81]. Moreover, new mechanism of cell death modulation has also been linked to lncRNA through interaction with protein factors, leading to apoptosis resistance [82]. However, the downfall of these findings is that the underlying mechanisms still remain largely unknown. Nonetheless, Divergent has been presented in this study to be consistently downregulated across tumors and seems to be associated with pivotal processes, involved in cancer growth and development; investigating its possible role in splicing events and cell growth may be of benefit.

#### 3.5.4. GO Terms Shared across Networks

We reported four processes to be shared by both lncRNAs (Figure 5). Interestingly, these processes were found in the network of negatively correlated mRNAs with Antisense, reported to be upregulated, and that of positively correlated mRNAs with Divergent, found to be downregulated in cancers. Taken together, it appears that although these two lncRNAs are interacting with different sets of mRNAs across different cancers, and possibly through different mechanisms with one being upregulated and the other shown to be downregulated; they are both enriching substantially similar processes, found to be fundamental in cancer proliferation and progression. 

### 3.6. Evaluation of lncRNA DE on Independent Datasets

**Figure 6:**
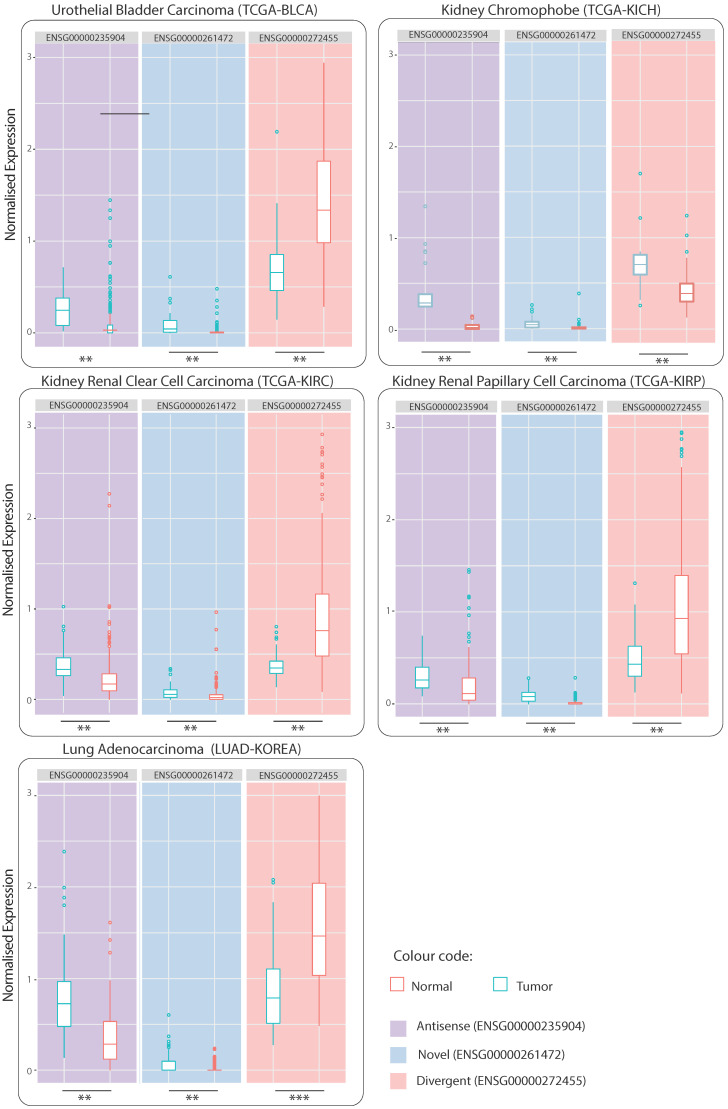
Boxplots of normalised expression values of lncRNAs Antisense, Novel and Divergent, in tumor versus non-neoplastic samples across five other cancer types which have not been used during the discovery phase of these lncRNAs. These include TCGA datasets, namely urothelial bladder carcinoma (BLCA), kidney chromophobe (KICH), kidney renal clear cell carcinoma (KIRC), and cervical kidney renal papillary cell carcinoma (KIRP), and one non-TCGA lung adenocarcinoma dataset collected in Seoul, South Korea (LUAD_KOREA). Significant adjusted *p*-values (adj *p* ≤ 0.01) are represented by a star, from left to right: the *t*-test and the Wilcoxon test.

We were interested to evaluate if our findings are generalisable to other cancer types not included in the discovery phase of the three lncRNAs. Accordingly, we assessed the dysregulation of Antisense, Novel, and Divergent on independent TCGA and non-TCGA datasets obtained from TANRIC repository. These include the total of 1047 tumor samples and their related adjacent 218 non-neoplastic tissue samples across five cancer subtypes, four of which are sourced from TCGA including urothelial bladder carcinoma, BLCA (19 non-neoplastic and 252 tumor samples), kidney chromophobe, KICH (25 non-neoplastic and 66 tumor), kidney renal clear cell carcinoma, KIRC (67 non-neoplastic and 448 tumor), and cervical kidney renal papillary cell carcinoma, KIRP (30 non-neoplastic and 198 tumor samples), and one is a non-TCGA dataset collected in Seoul, South Korea comprising 77 non-neoplastic and 83 tumor samples of lung adenocarcinoma (LUAD_KOREA) [83]. We investigated the DE of the three lncRNAs of interest across each dataset. In addition to the *t*-test, we estimated *p*-values using a non-parametric test (i.e., the two-sample Wilcoxon test or the Mann–Whitney test) to mitigate the biases associated with the underlying assumptions of hypothesis tests. The *p*-values were adjusted for multiple hypothesis testing using the Bonferroni correction. In agreement with our results, we observed the three lncRNAs to be consistently dysregulated across all five cancer types, with adjusted *p*-value ≤ 0.01 (Figure 6 and Table S6). While the direction of dysregulation can be different in the validation datasets compared to the discovery datasets, the significance of DE (independent of the hypothesis test) supports the potential role of these lncRNAs across diverse cancer types and provides more evidence that lncRNAs identified in this study could serve in potentially developing new pan-cancer biomarkers or therapeutic agents.

### 3.7. Future Direction

The foundational processes identified in this study, such as angiogenesis, underly all tumors regardless of tissue type. Understanding the role of lncRNAs in enriching these perturbations is thus very important and can be advantageous, particularly when malignant cells spread to other tissues, leading to current treatment strategies in becoming somewhat ineffective. A possible future direction would be to use other computational approaches and databases to decipher putative functions of lncRNAs. For instance, taking an integrative approach considering transcriptomic and epigenomic data (e.g., methylation profile [84]), or genomic changes such as copy number alterations (CNAs) [85]. In addition, using UCSC genome browser [86], we could also examine the genomic location of lncRNAs along with co-expressed coding genes in order to explore cis and trans-relationships, which can be an initial step in discovering potential regulatory roles. Further analysis of the dysregulation of the identified lncRNAs can also be performed across other datasets, allowing further evidence to be provided for their dysregulation across other cancer types. Moreover, we have focused in the present study on commonly dysregulated lncRNAs, the complimentary future approach could be to explore lncRNAs that are specific to each cancer type/subtype, with the aim of investigating cancer-specific functions, which can also be of benefit. 

## 4. Materials and Methods

### 4.1. Data Source

We used TANRIC to access expression data of lncRNAs of 3326 tumor samples and their related adjacent 416 non-neoplastic tissue samples for eight cancer subtypes as categorised in TCGA. Included were 105 non-neoplastic and 837 tumor samples of breast invasive carcinoma (BRCA), 58 non-neoplastic and 488 tumor samples of lung adenocarcinoma (LUAD); 17 non-neoplastic and 220 tumor samples of lung squamous cell carcinoma (LUSC); 52 non-neoplastic and 374 tumor samples of prostate adenocarcinoma (PRAD); 59 non-neoplastic and 497 tumor samples of thyroid carcinoma (THCA); 33 non-neoplastic and 285 tumor samples of stomach adenocarcinoma (STAD), 50 non-neoplastic and 200 tumor samples of liver hepatocellular carcinoma (LIHC); and 42 non-neoplastic and 425 tumor samples of head and neck squamous cell carcinoma (HNSC). An overview of the workflow of this study is shown in Figure S4.

### 4.2. Differential Exprssion Analysis and Commonality Exploration

We carried out differential expression analysis by comparing lncRNA expression levels between tumor and related adjacent non-neoplastic samples of a given set. Expression data for a total of 12,727 lncRNAs was downloaded from TANRIC v2.0 for each of the eight TCGA cancer types. Differentially expressed lncRNAs (up- and downregulated) were identified (|log_2_(FC)| > 1) and Student’s *t*-test was applied to calculate *p*-values followed by the Benjamini and Hochberg method to control the false discovery rate (FDR) [87]. Differentially expressed lncRNAs with adjusted *p*-value ≤ 0.01 were considered statistically significant. We then identified common dysregulated lncRNAs (found in 2+ cancers) and unique dysregulated lncRNAs (specific to a given cancer type). Commonality was evaluated between each pair of cancers and represented by the Jaccard index (*J*) [88]. These analyses were implemented in MATLAB with the code available on the GitHub repository (https://github.com/VafaeeLab/PanCancer-lncRNAs). 

#### 4.2.1. mRNA Correlation Analysis

Guided by the “guilt by association” principle [89], we utilised TANRIC to explore mRNAs correlated with common dysregulated lncRNAs across each cancer type. Spearman rank correlation coefficient (rs) were calculated to examine correlation relationships between lncRNAs of interest and mRNAs expression. Lists of correlated mRNAs were extracted based on cut-offs of rs ≥ 0.5 or rs ≤ −0.5, for positive and negative correlation, respectively, with correlation *p*-value ≤ 0.01.

#### 4.2.2. Functional Enrichment Analysis

Exploration of large sets of genes can be achieved by organising them based on common functional features and one of the most widely used ways to understand genes and their products is to explore gene ontologies (GO) [90,91]. Thus, to investigate functional implications of lncRNAs of interest, we performed GO enrichment analysis with particular focus on biological processes. We exploited WebGestalt (WEB-based gene set analysis toolkit), to identify GO terms enriched by mRNAs lists, found to be correlated with common differentially expressed lncRNAs, with the aim of perusing their functional role [92,93]. Statistical analysis of GO enrichment was performed using a Fisher’s exact test with a hypergeometric null distribution [94,95]. Significantly enriched GO terms were determined as FDR ≤ 0.05.

#### 4.2.3. GO Similarity Analysis

GO, considered as a universal vocabulary, is structured as a hierarchical directed acyclic graph where each node represents a class of gene function (GO term), and the connection between two GO terms indicates different relationships. This hierarchy allows exploring semantic similarities among enriched GO terms, which could imply functional similarities between the associated genes [61,96]. Following the annotation of mRNAs lists by ontology, we merged GO terms enriched by each lncRNA across different cancers and distinguished two separate groups: GO terms enriched by positively and negatively correlated genes. After additional filtering, based on FDR ≤0.05, GO terms were then further investigated. In order to measure closeness of GO terms using NaviGO, an interactive software that allows the retrieval of functional similarity scores and visualisation as networks [97]. From the six different scoring schemes offered by NaviGO, we relied on Relevance Semantic Similarity (RSS), which measures the relative depth and rareness of the biological processes involved [61]. RSS ranges from 0 to 1, with 0 representing zero similarity and 1 indicating very high similarity. Functional similarity networks were created using the NaviGO visualiser based on threshold RSS ≥ 0.5.

## 5. Conclusions

Once seen as transcriptional by-products, lncRNAs are now emerging as key players in cellular function, regulating a wide range of biological processes, and involved in their disruption. Our study provides evidence that lncRNAs may be contributing to the hallmarks of cancer, regardless of cancer type. These results are promising, suggesting that lncRNAs can serve as potential therapeutic targets to be applied in multiple cancer subtypes; future treatment strategies may potentially include non-coding genes in addition to specific protein targets. Indeed, lncRNAs have brought a promising new era to cancer biology, especially in terms of diagnosis and therapy.

## Data Availability

Data used in this study are publicly accessible via TANRIC, The Atlas of non-coding RNA in Cancer (www.tanric.org). Codes generated in this study are available at the GitHub repository https://github.com/VafaeeLab/PanCancer-lncRNAs.

## Supplementary Materials

The following supporting information can be downloaded at: https://www.mdpi.com/article/10.3390/ijms24054796/s1.
