# Supplementary material for: Pan-Cancer Analysis Reveals Functional Similarity of Three lncRNAs across Multiple Tumors
Source: Int J Mol Sci. 2023 Mar 1;24(5):4796. doi: 10.3390/ijms24054796 (PMC10003012; doi:10.3390/ijms24054796)
Supplement: Supplementary file 1 [file ijms-24-04796-s001.zip › ijms-2237778-Supplementary.pdf]

Pan-Cancer Analysis Reveal Functional Similarity of Three lncRNAs across Multiple Tumors

Abir Khazaal [1], Seid Miad Zandavi [1,2], Andrei Smolnikov [1], Shadma Fatima [1,3], and Fatemeh Vafaee [1,4,*]

[1] School of Biotechnology and Biomedical sciences, Faculty of Science, University of New South Wales, Sydney NSW 2052, Australia
[2] Harvard Medical School, Harvard University, Boston, MA, USA
[3] Ingham Institute of Applied Medical Research, Sydney NSW 2170, Australia
[4] UNSW Data Science Hub, University of New South Wales, Sydney NSW 2052, Australia
* Correspondence: f.vafaee@unsw.edu.au

List of Supplementary Figures and Tables:

Figure S1. **Heatmap representing commonality of lncRNAs between different pairs of cancers**. Similarity scores amid pairs of cancers indicated by Jaccard index. *J* scores range from 0-1 with white shaded boxes (J=0) indicating low similarity and black shaded boxes (J=1) indicating very high similarity (identical sets of lncRNAs).

Figure S2. **Summary of gene ontologies (FDR ≤ 0.05) enriched across all cancer types by each lncRNA: Antisense, Novel and Divergent**. GO terms grouped by the nature of relationship with respective lists of mRNAs; positive and negative correlation.

Figure S3. **Summary of Semantic similarity scores of GO terms enriched by both lncRNAs "Antisense" and "Divergent".** Heatmap representing semantic similarity scores of GO terms enriched by all sets of mRNAs (combined), positively and negatively correlated (when applicable) with lncRNAs Antisense and Divergent. Blue indicates low similarity and red indicates high similarity. Three blocks can be seen. Three clusters can be identified in the first block, named Antisense Network (+). Shared GO terms between second block/Antisense Network (-) and third block/Divergent Network (+) are bolded.

Figure S4. Overview of analyses performed in this study. The arrows show where data from an analysis has been used in the ensuing analysis.

Table S1. Lists of differentially expressed lncRNAs (|log2(FC)| > 1 and FDR ≤ 0.01), comparing tumors with adjacent normal samples) identified across each cancer.

Table S2. Distribution of differentially expressed lncRNAs (upregulated and downregulated) across TCGA cancer types.

Table S3. Table showing details on lncRNAs found to be dysregulated across all cancer types.

Table S4. $\log_2$(Fold change) and FDR values of three lncRNAs, consistently dysregulated across all cancers.

Table S5. Lists of correlated mRNAs ($|rs| \geq 0.5$ and p-value ≤ 0.01) with three consistently dysregulated lncRNAs, across each cancer type**.**

Table S6. Lists of correlated mRNAs ($|rs| \geq 0.5$ and p-value ≤ 0.01) with three consistently dysregulated lncRNAs, across each cancer type**.**

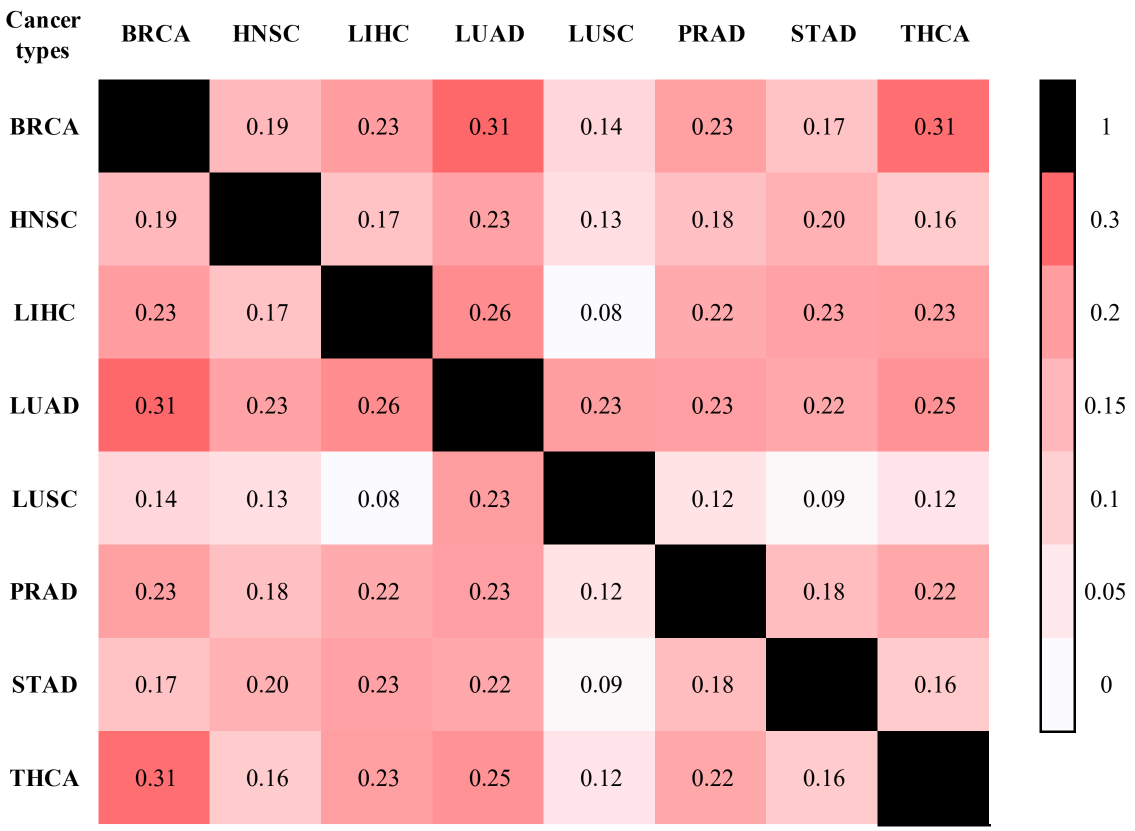

Figure S1. Heatmap representing commonality of lncRNAs between different pairs of cancers. Similarity scores amid pairs of cancers indicated by Jaccard index. *J* scores range from 0-1 with white shaded boxes (J=0) indicating low similarity and black shaded boxes (J=1) indicating very high similarity (identical sets of lncRNAs).

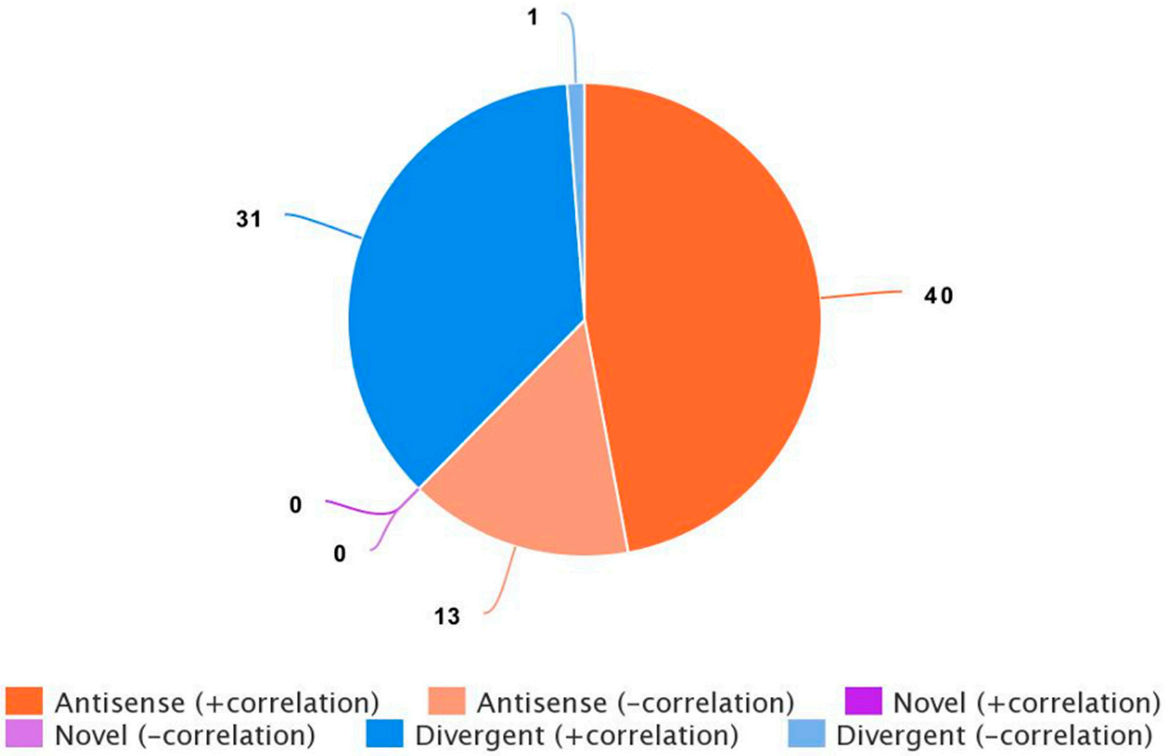

Figure S2. **Summary of gene ontologies (FDR ≤ 0.05) enriched across all cancer types by each lncRNA: Antisense, Novel and Divergent**. GO terms grouped by the nature of relationship with respective lists of mRNAs; positive and negative correlation.

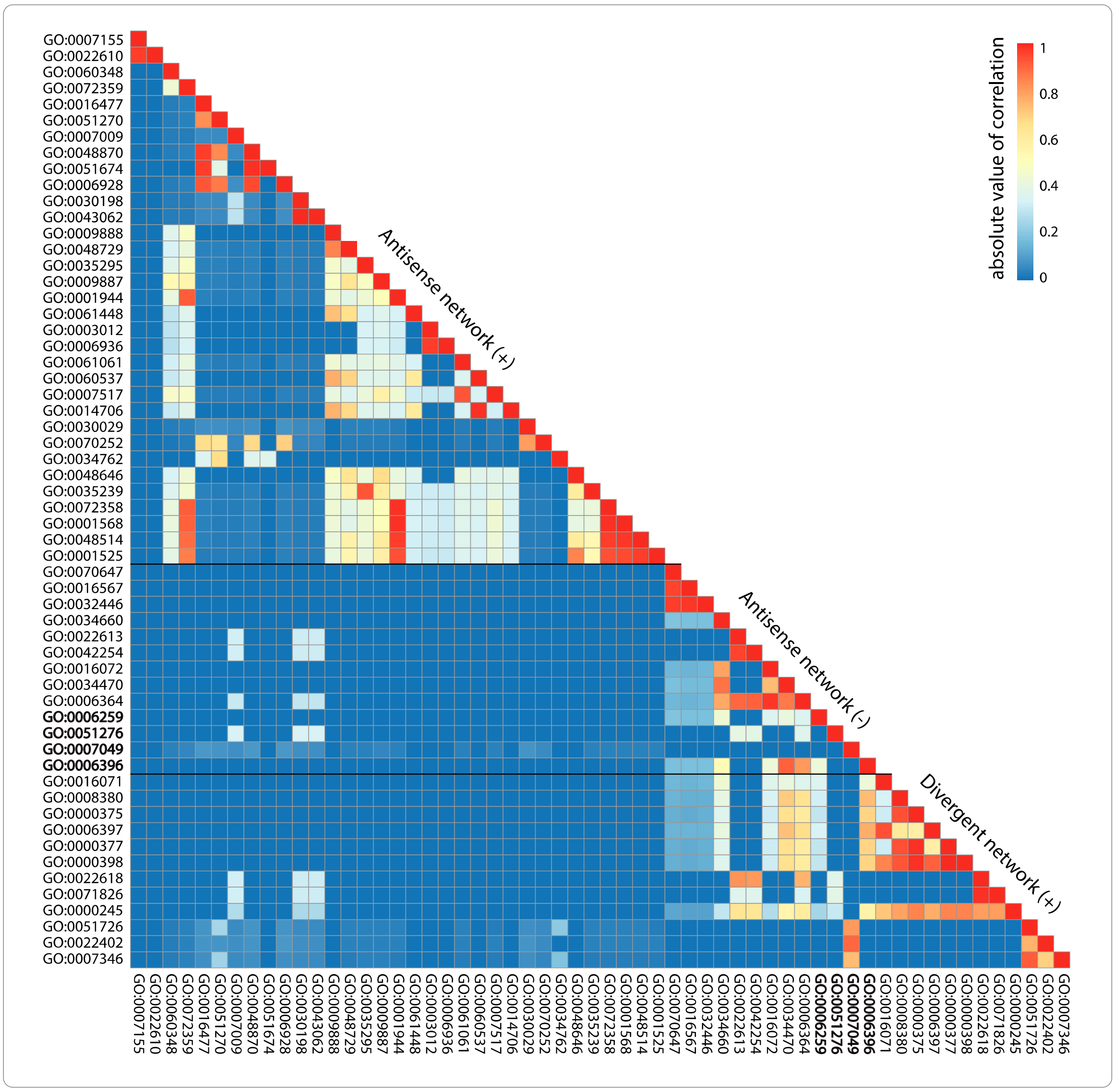

Figure S3. **Summary of Semantic similarity scores of GO terms enriched by both lncRNAs "Antisense" and "Divergent".** Heatmap representing semantic similarity scores of GO terms enriched by all sets of mRNAs (combined), positively and negatively correlated (when applicable) with lncRNAs Antisense and Divergent. Blue indicates low similarity and red indicates high similarity. Three blocks can be seen. Three clusters can be identified in the first block, named Antisense Network (+). Shared GO terms between second block/Antisense Network (-) and third block/Divergent Network (+) are bolded.

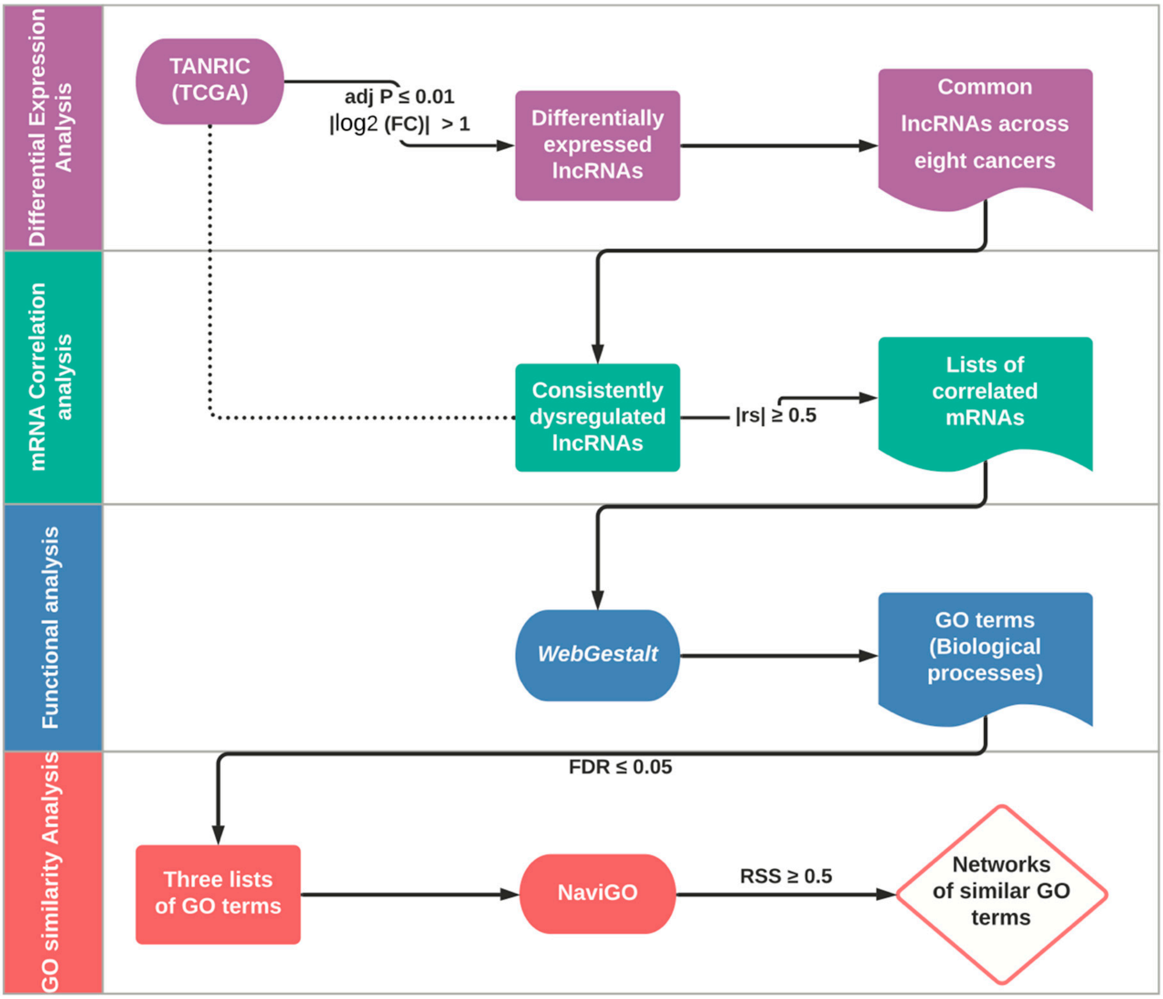

Figure S4. Overview of analyses performed in this study. The arrows show where data from an analysis has been used in the ensuing analysis.

Table S1. Lists of differentially expressed lncRNAs (|log2(FC)| > 1 and FDR ≤ 0.01, comparing tumors with adjacent normal samples) identified across each cancer.

[Attached as an Excel Sheet]

Table S2. Distribution of differentially expressed lncRNAs (upregulated and downregulated) across TCGA cancer types.

**Cancer**	**Breast (BRCA)**	**Throat (HNSC)**	**Liver (LIHC)**	**Lung (LUAD)**	**Lung (LUSC)**	**Prostate (PRAD)**	**Stomach (STAD)**	**Thyroid (THCA)**
N. of differentially expressed lncRNA	4807	2098	3024	3747	1212	2653	2101	4861
% of upregulated lncRNAs	69.8%	50%	5.8%	47.8%	93.8%	57.3%	21.3%	81%
% of downregulated lncRNAs	30.2%	50%	94.2%	52.2%	6.2%	42.7%	78.7%	19%
% of common lncRNAs	84.6%	90.9%	92.4%	92%	97.3%	92%	91.2%	80.3%

Table S3. Table showing details on lncRNAs found to be dysregulated across all cancer types.

Ensembl ID	*Gene Name*	*Annotation*	*Location (GRCh38)*	*Strand*	*Transcripts*
ENSG00000223561.7	Novel	TAGENE	Chr7: 25,593,304-25,751,032	reverse	25
ENSG00000257167.2	TMPO-AS1	Manual (Havana project)	Chr12: 98,512,973-98,516,422	reverse	2
ENSG00000249859.12	PVT1	Manual (Havana project)	Chr8: 127,794,526-128,187,101	forward	176
ENSG00000245522.2	LINC02709	Manual (Havana project)	Chr11: 9,754,770-9,759,533	reverse	2
ENSG00000235904.3	RBMS3-AS3	Manual (Havana project)	Chr3: 29,054,570-29,290,726	reverse	5
ENSG00000261472.1	Novel	Manual (Havana project)	Chr16: 79,505,603-79,516,293	forward	1
ENSG00000272455.1	MRPL20-DT	Manual (Havana project)	Chr1: 1,409,096-1,410,618	forward	1

Table S4. $\log_2$(Fold change) and FDR values of three lncRNAs, consistently dysregulated across all cancers.

**Cancer**	**ENSG00000235904 Antisense**		**ENSG00000261472 Novel**		**ENSG00000272455 Divergent**	
	**$\log_2$ FC**	**FDR**	**$\log_2$ FC**	**FDR**	**$\log_2$ FC**	**FDR**
**BRCA**	3.45	4.61E-100	3.48	4.01E-105	-1.91	4.39E-06
**HNSC**	1.69	3.47E-04	2.51	4.69E-17	-3.21	5.20E-05
**LIHC**	1.64	6.88E-03	1.61	9.34E-03	-4.15	6.81E-04
**LUAD**	1.91	3.57E-09	2.46	3.09E-22	-3.11	4.32E-07
**LUSC**	2.41	6.87E-07	2.28	1.15E-05	-4.11	6.89E-03
**PRAD**	2.41	6.22E-29	1.40	2.37E-03	-2.16	3.85E-06
**STAD**	2.40	3.50E-09	1.64	7.28E-03	-3.64	1.94E-03
**THCA**	1.40	5.77E-05	1.33	5.22E-04	-1.69	7.50E-06

Table S5. Lists of correlated mRNAs (|rs| ≥ 0.5 and *p*-value ≤ 0.01) with three consistently dysregulated lncRNAs, across each cancer type**.**

[Attached as an Excel Sheet]

Table S6. Lists of correlated mRNAs (|rs| ≥ 0.5 and *p*-value ≤ 0.01) with three consistently dysregulated lncRNAs, across each cancer type**.**

Cancers	Statistical test	ENSG00000235904	ENSG00000261472	ENSG00000272455
		adj p-value	adj p-value	adj p-value
BLCA	t-test	2.31E-02	4.04E-02	3.65E-08
	Wilcoxon	4.88E-06	4.27E-07	1.39E-07
KICH	t-test	3.79E-06	1.36E-02	1.41E-02
	Wilcoxon	7.25E-13	2.42E-05	1.37E-05
KIRC	t-test	2.31E-08	2.56E-03	5.77E-31
	Wilcoxon	3.02E-14	5.53E-06	1.51E-21
KIRP	t-test	2.67E-02	4.44E-05	1.97E-13
	Wilcoxon	1.92E-04	2.81E-11	6.71E-07
LUAD_KOREA	t-test	1.33E-10	8.31E-03	8.34E-14
	Wilcoxon	6.43E-12	6.64E-03	2.19E-14
